# Genetic Basis of Fiber Improvement and Decreased Stress Tolerance in Cultivated Versus Semi-Domesticated Upland Cotton

**DOI:** 10.3389/fpls.2019.01572

**Published:** 2019-11-29

**Authors:** Guozhong Zhu, Weixi Li, Guilin Wang, Lechen Li, Qingxin Si, Caiping Cai, Wangzhen Guo

**Affiliations:** State Key Laboratory of Crop Genetics and Germplasm Enhancement, Cotton Hybrid R & D Engineering Center (the Ministry of Education), Nanjing Agricultural University, Nanjing, China

**Keywords:** upland cotton, single nucleotide polymorphism array, expression profile, domestication, artificial selection, fiber development, stress tolerance

## Abstract

Crop domestication from wild ancestors has resulted in the wide adaptation coupled with improved yield and quality traits. However, the genetic basis of many domesticated characteristics remains to be explored. Upland cotton (*Gossypium hirsutum*) is the most important tetraploid cotton species, accounting for about 90% of world cotton commerce. Here, we reveal the effects of domestication on fiber and stress traits through comprehensive analyses of semi-domesticated races and cultivated cotton accessions. A total of 416 cotton accessions were genotyped, and a decrease in genetic diversity from races to landraces and modern cultivars was detected. Furthermore, 71 domestication selective sweeps (DSS) and 14 improvement selective sweeps (ISS) were identified, with the Dt sub-genome experiencing stronger selection than the At sub-genome during the both selection types. The more expressed genes and a delay in the expression peak of genes related to secondary cell wall (SCW) development in modern cultivars compared to semi-domesticated cotton races, may have contributed to long fibers in these plants. However, down-regulation of genes related to stress response was responsible for decreasing stress tolerance in modern cultivars. We further experimentally confirmed that silencing of *PR1* and *WRKY20*, genes that showed higher expression in the semi-domesticated races, drastically compromised cotton resistance to *V. dahliae*. Our results reveal fiber improvement and decreased stress tolerance as a result of the domestication of modern upland cotton cultivars.

## Introduction

Crop domestication of wild plants has resulted in a variety of physiological and economical improvements. Cotton (*Gossypium* spp.) is the foremost natural fiber and oil source in the world, and allopolyploid cottons have been the subject of evolutionary and domestication investigations into the genomic mysteries of polyploidy (Adams and Wendel, 2004; Chen et al., 2007). The allotetraploids that were present 1–2 million years ago (MYA) originated from one hybridization event between an extinct progenitor of *Gossypium herbaceum* (A1) or *Gossypium arboreum* (A2) and another progenitor, *Gossypium raimondii* (D5) (Wendel, 1989). Originally native to Mexico-Guatemala and the Caribbean, upland cotton was first domesticated at least 4,000–5,000 years ago, and was subsequently subjected to directional selection (Wendel et al., 1992). In later evolution and domestication studies, upland cotton was often divided into three groups; wild or semi-domesticated races (also named semi-wild), landraces, and improved modern cultivars (Fang et al., 2017a; Fang et al., 2017b; Wang et al., 2017). There are seven races of *G. hirsutum* that are presently recognized as wild or semi-wild, named as “*yucatanense*,” “*punctatum*,” “*palmeri*,” “*latifolium*,” “*mariegalante*,” “*morrilli*,” and “*richmondi*,” respectively (Hutchinson, 1951). Further, several localized derivatives of the domesticated accessions are referred to as founders or landraces. The improved modern cultivars that are widely cultivated in nowadays and have a high fiber yield and superior fiber quality, are mainly derived from the landraces.

Modern improved varieties of *G. hirsutum* (“upland cotton”), which is considered to be the most important tetraploid cotton species, are widely cultivated in over 50 countries and account for about 90% of the world’s cotton commerce (Wendel et al., 2012). Compared to perennial ancestors with shorter and sparser fibers, modern improved upland cotton is annual, day-length neutral, high-yielding plants with easily ginned, good quality, and abundant fibers (Wendel et al., 1992). However, the domestication process is accompanied by an extreme reduction in genetic diversity (Fang et al., 2017a; Fang et al., 2017b; Wang et al., 2017). The narrow genetic base of improved upland cotton has become a serious concern, since limited genetic diversity leads to limited allelic availability for continued genetic gain (Brown, 1983). It is important to understand the genetic basis of domesticated traits and to maintain species diversity for future improvement of upland cotton.

Studies on the genetic diversity and domestication of upland cotton have increased rapidly since the advent of molecular marker technology (Brubaker and Wendel, 1994; Tyagi et al., 2014; Hinze et al., 2016). Benefiting from the development of high-throughput sequencing, the genome of upland cotton has been well assembled and annotated (Li et al., 2015; Zhang et al., 2015; Hu et al., 2019; Wang et al., 2019), which provides a good foundation for follow-up genome-wide studies on the evolution and domestication. Recent genome resequencing analyses revealed the population structure and evolutionary characteristics of upland cotton and identified key loci or genes associated with fiber development during artificial selection (Fang et al., 2017a; Fang et al., 2017b; Wang et al., 2017; Ma et al., 2018). In addition, transcriptome and protein sequencing has also revealed important genetic changes associated with the evolution of cotton (Applequist et al., 2001; Hovav et al., 2008a; Rapp et al., 2010; Hu et al., 2011; Hu et al., 2013). However, the reported studies mainly focused on the improvement of fiber development, there are few on other domesticated traits such as stress tolerance. Stress tolerance is an important trait for cotton productivity. Breeding of crops over millennia for yield and productivity has led to reduced genetic diversity. As a result, beneficial traits of wild species, such as disease resistance and abiotic stress tolerance, have been lost in modern cultivars. A previous RNA-seq study suggests that wild cottons allocate greater resources to stress response pathways than modern cultivars, while domestication has led to reprogrammed resource allocation toward increased fiber growth (Yoo and Wendel, 2014). Crop plants are often grown under unfavorable environmental conditions that prevent the full expression of their genetic potential for productivity. The average yield of major U.S. crops (corn, wheat, soybean, sorghum, oat, barley, potato, and sugar beet) is only 21.6% of the highest yield attained (Boyer, 1982). In cotton, the world average yield is estimated at 650 kg lint ha^−1^, with around 73% of potential yield lost to various stresses. For example, soil salinity ranging from 8 to 18 dSm^−1^ resulted in yield losses of 15–55% in cotton (Satir and Berberoglu, 2016). The large number of wild *Gossypium* species presents an impressive range of variation in many characteristics, such as high fiber strength, disease resistance, nectariless, and glandless traits, all of which are potentially available for exploitation in cotton improvement programs (Wendel et al., 2009). Several stress-tolerance associated QTLs have been excavated in modern cultivated cotton, however, the excellent stress-tolerance loci still remain unknown in wild cotton (Majeed et al., 2019). Therefore, mining and restoring crop plant alleles from wild ancestors that were ‘‘left behind’’ during domestication may be useful for the improvement of productivity under stress conditions in modern cotton breeding.

*G. hirsutum* is an attractive model for elucidating the genetic changes of domesticated traits due to its evolutionary history of semi-wild, landrace, and modern cultivars. In this study, we genotyped 416 cotton accessions using the CottonSNP80K array, comprising *G. hirsutum* semi-wild, landrace, and modern cultivars, with tetraploid wild cotton species and *G. barbadense* cotton accessions as outgroups. Further, population genomic analysis was conducted and selective sweeps were identified. Integrated transcriptome and functional analyses showed that domestication and artificial selection of *G. hirsutum* contributed to fiber improvement and decreased stress tolerance, and the semi-domesticated races can act as an important genetic source for improved stress tolerance in modern upland cotton cultivars.

## Materials and Methods

### Plant Materials

A total of 416 diverse cotton accessions were collected, comprising of 5 tetraploid wild accessions, 16 sea island cotton (*G. barbadense*) accessions, 56 semi-domesticated upland cotton accessions (involved in seven races), 31 upland cotton landraces, and 308 modern cultivated upland cotton accessions (**Supplementary Table S1**).

*G. hirsutum* acc. TM-1, a genetic standard line of upland cotton, was used for the salt, drought and *V. dahliae* stress treatments, and RNA-sequencing analysis. Disease-resistant cultivar *G. barbadense* cv. Hai7124 was used for the virus-induced gene silencing (VIGS) study, with disease-susceptible *G. hirsutum* acc. TM-1 as control.

### Genotyping Analysis

Genomic DNA was extracted from cotton leaves *via* the CTAB method (Paterson et al., 1993). A CottonSNP80K array containing 77,774 single nucleotide polymorphisms (SNPs) was used to genotype the 416 accessions (Cai et al., 2017). Qualified DNA, which showed single DNA band on 1% agarose gel electrophoresis with no diffusion, and no RNA residue, and exhibited light absorption value A260/280 between 1.7 and 2.1, was hybridized to the array following the Illumina protocols. The Illumina iScan array scanner was used to scan arrays, and GenomeStudio Genotyping software (V2011.1, Illumina, Inc.) was employed to cluster single nucleotide polymorphism (SNP) alleles and genotyping with a manual corrected clustering file (Cai et al., 2017). The SNP data set with a calling rate < 0.9 and minor allele frequency (MAF) < 0.05 was further filtered, and high quality data was used for subsequent analysis. The SNPs were annotated with referring *G. hirsutum* acc. TM-1 genome sequences (Zhang et al., 2015).

### Population Properties and Linkage Disequilibrium Analysis

PLINK V1.90 software (Purcell et al., 2007) was employed to conduct similarity analysis of 416 cotton accessions. Based on the distance matrix data, phylogenetic trees were constructed with a neighbor-joining method using Tassel 5.0 software (Bradbury et al., 2007), and visually edited by FigTree software (http://tree.bio.ed.ac.uk/software/figtree/). Principal component analysis (PCA) was performed using Tassel 5.0 software and visually edited by R software. Population structure analysis was performed using admixture 1.3 software (Alexander et al., 2009). The correlation coefficient (*r* ^2^) of alleles was calculated to measure linkage disequilibrium (LD) in each upland cotton group level using PLINK V1.90.

### Detection of Artificial Selection Regions

A composite scoring system was used to determine whether a sliding window was under selection. In order to ensure each sliding window had at least two SNPs, we counted the SNP distances and carried out a gradient simulation with different window sizes. Nucleotide diversity (π) and population divergence (*F*st) between pairwise upland cotton groups were calculated in each sliding window by Vcftools (Danecek et al., 2011). The domestication selective sweeps (DSS) were identified by the overlap of the top 5% of the π ratio and *F*st value and the improvement selective sweeps (ISS) were identified by the overlap of the top 1% of the π ratio and *F*st value. To explore the evolutionary genetics of cotton fiber domestication, we overlapped selective sweeps with the locations of genome-wide association analysis (GWAS) loci reported previously (Fang et al., 2017b; Sun et al., 2017; Wang et al., 2017; Ma et al., 2018). The shell script written by ourselves was used for extracting genes in the selective sweeps, with *G. hirsutum* acc. TM-1 genome sequences (Zhang et al., 2015) as the reference.

### RNA-Seq Data Analysis

The RNA-seq data of two semi-wild cotton relatives (*G. hirsutum* race. *palmeri*; *G. hirsutum* race. *yucatanense*) were downloaded from the National Center for Biotechnology Information (NCBI) Sequence Read Archive PRJNA178969 for expression analysis of fiber developmental genes (Paterson et al., 2012). The RNA-seq data of 50 semi-wild and cultivated cottons were downloaded from NCBI Sequence Read Archive collection SRP080913 for expression analysis of stress responsive genes (Wang et al., 2017). The RNA-seq data in different TM-1 tissues were downloaded from NCBI Sequence Read Archive collection SRP044705 (Zhang et al., 2015).

For the salt and drought treatments, *G. hirsutum* acc. TM-1 seedlings were irrigated with different concentrations of NaCl (100, 150, 200, 250, 300 mM) and PEG (6000) (5%, 10%, 15%, 20%, 25%) for determination of optimization stress concentration, respectively. We detected the obvious stress phenotype under 200 mM NaCl and 15% PEG treatments, respectively, with slight harm to plant growth and development. So, 200 mM NaCl and 15% PEG were selected for stress-induced expression analysis, with untreated seedlings as control. For *V. dahliae* treatment, *G. hirsutum* acc. TM-1 seedlings were dipped in *V. dahliae* strain V991 conidial suspensions containing 1×10^7^ spores mL^-1^ (untreated seedlings as control). Treated roots and control check (CK) were sampled at the appropriate time points for gene expression analysis. Total RNA was isolated using the CTAB method (Liu et al., 2006). RNA sequencing was performed on an Illumina HiSeq 2000 system. After pre-processing the RNA-seq data with an NGS QC toolkit (Patel and Jain, 2012), the reads were mapped to the *G. hirsutum* acc. TM-1 genome using a Tophat spliced aligner with default parameters (Trapnell et al., 2009). The genome-matched reads from each library were assembled with Cufflinks (Trapnell et al., 2012). Cuffmerge was then used to merge the individual transcript assemblies into a single transcript set. Lastly, Cuffdiff was used to detect differentially expressed genes (DEGs) with a cutoff of 0.05 *q*-value. Three biological replicates from each sample were used for these RNA-seq experiments. These RNA-seq data have been deposited in the NCBI database under BioProject accession PRJNA532694.

### Quantitative Real Time PCR Analysis

Quantitative real time PCR (qRT-PCR) was performed using a Bio-Rad CFX96 Real-Time instrument and the light cycler fast start DNA Master SYBR Green I kit (Roche, Basel, Switzerland). Reactions were performed with three technical replicates for each biological sample, and contained 100 ng of cDNA, 0.5 µl of each primer (10 µM/µl), and 10 µl SYBR Green Master Mix in a final volume of 20 µl. The amplification was performed under the following conditions: 95°C for 5 min, followed by 40 cycles of 95°C for 15 s, 58°C for 20 s, and 72°C for 30 s. Melting curve analysis, performed by increasing the temperature from 55°C to 95°C (0.5°C per 10 s), and agarose gel electrophoresis of the final product confirmed the presence of single amplicons. Relative fold diﬀerences for each sample in each experiment were calculated using the 2^−ΔCt^ method (Livak and Schmittgen, 2001). The *GhHis3* gene was used as a control. Expression data from three biologically independent experiments were analyzed and presented as means ± S.D. Primer pairs for qRT-PCR are shown in **Supplementary Table S2**.

### VIGS Experiments

We constructed TRV: *GbPR1*, TRV: *GbWRKY20*, and TRV: *GhCLA1* vectors for VIGS analysis. Primer pairs to generate TRV vectors were shown in **Supplementary Table S2**. These vectors were transformed into *A. tumefaciens* strain GV3101 (Clough and Bent, 1998). Subsequently, cotyledons of 7-day-old Hai7124 cotton seedlings were respectively infiltrated with 1:1 mixtures of pTRV1 and pTRV constructs as described previously (Xu et al., 2017). All plants were grown in the same growth chamber at 23/21°C (day/night), with a 16-h light/8-h dark cycle, and changes in plant phenotypes were observed.

About 2 weeks after *A. tumefaciens* inoculation, the TRV: *GhCLA1* plants showed highly uniform bleaching in newly emerged leaves. We harvested the roots from at least three seedlings per treatment and extracted the RNA for detecting the expression of target genes. All VIGS cotton seedlings were removed from the soil and dip-inoculated with V991 conidia suspension (1 × 10^7^ conidia/mL), with the susceptible TM-1 and the resistant Hai7124 as controls. All plants were grown in the same growth chamber at 25/23°C (day/night), with a 16 h light/8 h dark cycle for 25 days. The ratio of diseased to healthy leaves was investigated every five days after 10 days of inoculation. The VIGS experiments were repeated at least three times with at least 30 plants of each treatment.

## Results

### Genotyping Analysis

A total of 416 diverse cotton accessions were genotyped using the CottonSNP80K array. The genotype data revealed that these cotton accessions possessed a high average call rate of 99.39%. With low-quality (call rate < 90% and minor allele frequency < 0.05) loci filtered, a final set of 60,243 polymorphic SNPs was obtained, with 33,133 and 27,110 SNPs in the At and Dt sub-genomes, respectively (**Table 1** and **Supplementary Figure S1**). These SNP markers were distributed across the entire genome, with SNP density ranging from 19.20 to 55.67 SNP/kb. The polymorphism information content (PIC) values ranged from 0.32 to 0.41 among chromosomes, and the mean PIC was 0.37 in both the At and Dt sub-genomes, indicating high polymorphism of the SNPs selected on the array. The average PICs were 0.36, 0.35 and 0.33 in the semi-wild, landrace and modern cultivated cotton groups, respectively.

**Table 1:** Information on high quality SNPs used for genomic analysis.

Chr.	Number of SNPs	Chr. length (Mb)	Density of SNP (kb/SNP)	Call rate	MAF	Heterozygosity	PIC
A01	2525	99.88	39.56	0.99	0.30	0.23	0.39
A02	1499	83.45	55.67	0.99	0.28	0.19	0.38
A03	1903	100.26	52.69	0.99	0.29	0.19	0.38
A04	1141	62.91	55.14	0.99	0.29	0.19	0.38
A05	2683	92.05	34.31	0.99	0.28	0.17	0.38
A06	3229	103.17	31.95	0.99	0.25	0.24	0.34
A07	2308	78.25	33.90	0.99	0.28	0.19	0.37
A08	5472	103.63	18.94	0.99	0.26	0.26	0.35
A09	2635	75.00	28.46	0.99	0.27	0.20	0.36
A10	2174	100.87	46.40	0.99	0.26	0.20	0.36
A11	2212	93.32	42.19	0.99	0.26	0.18	0.35
A12	2172	87.48	40.28	0.99	0.27	0.18	0.36
A13	3180	79.96	25.14	0.99	0.28	0.20	0.38
At subgenome	33133	1160.23	35.02	0.99	0.28	0.20	0.37
D01	1942	61.46	31.65	0.99	0.29	0.17	0.38
D02	2495	67.28	26.97	0.99	0.30	0.17	0.39
D03	1458	46.69	32.02	0.99	0.23	0.18	0.32
D04	1084	51.45	47.46	0.99	0.29	0.16	0.38
D05	1715	61.93	36.11	0.99	0.27	0.17	0.36
D06	3349	64.29	19.20	0.99	0.26	0.19	0.36
D07	2854	55.31	19.38	0.99	0.27	0.17	0.37
D08	2385	65.89	27.63	0.99	0.33	0.16	0.41
D09	2485	51.00	20.52	0.99	0.25	0.17	0.34
D10	1823	63.37	34.76	0.99	0.28	0.15	0.37
D11	1602	66.09	41.25	0.99	0.27	0.15	0.36
D12	2116	59.11	27.93	0.99	0.27	0.19	0.36
D13	1802	60.53	33.59	0.99	0.26	0.15	0.36
Dt subgenome	27110	774.40	28.57	0.99	0.27	0.17	0.37

### Population Properties and Linkage Disequilibrium

**Figure 1:**
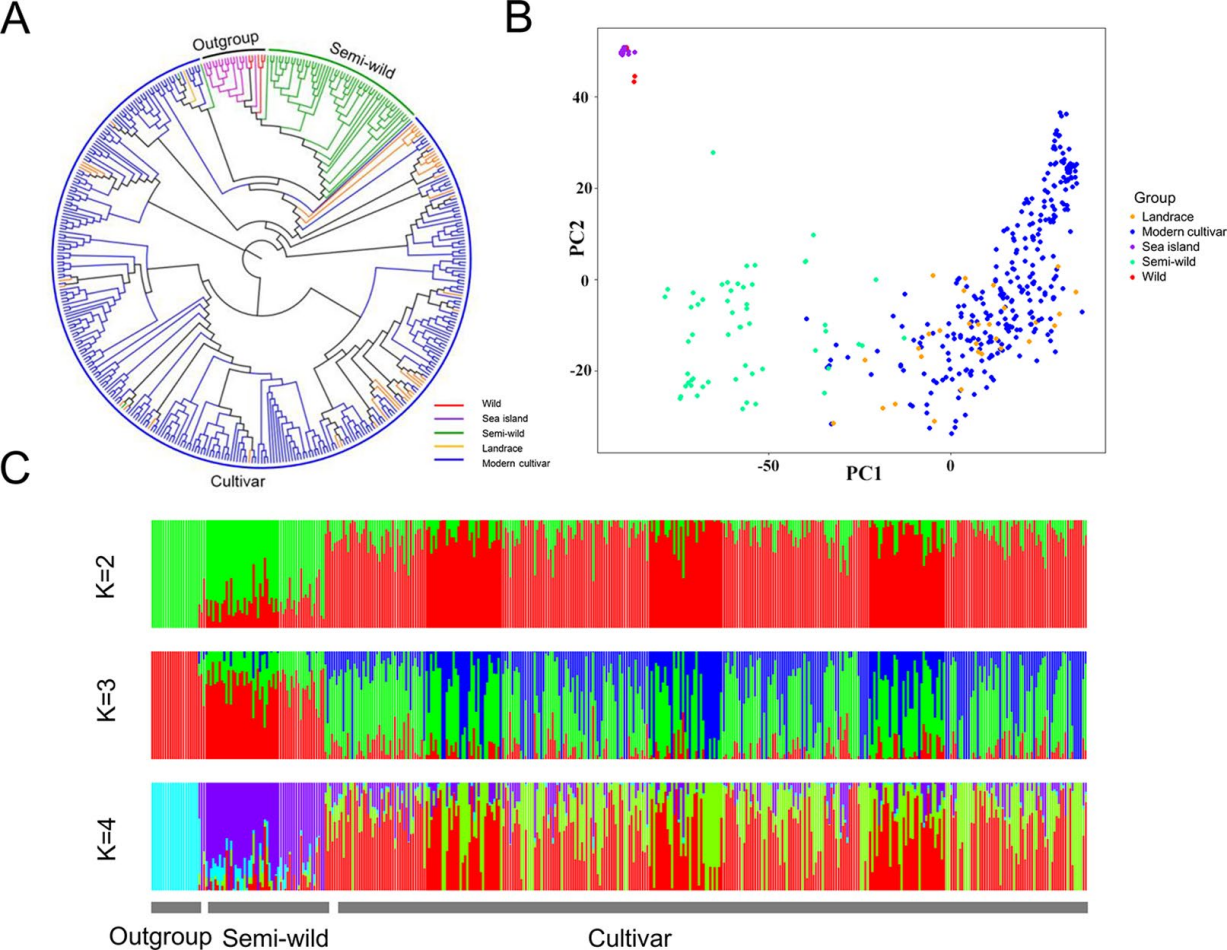
Phylogenetic relationships of 416 cotton accessions. **(A)**. A neighbor-joining tree was constructed using SNP data. **(B)**. PCA plot of the first two components (PC1 and PC2). **(C)**. Structure analysis with K  =  2, 3 and 4. The *y* axis quantifies cluster membership, and the *x* axis represents the different accessions.

The phylogenetic relationship showed that the 416 cotton accessions were clustered into three groups (**Figure 1A**), as supported by a PCA (**Figure 1B**). Model-based analyses of population structure using ADMIXTURE (**Figure 1C**) revealed that there were two different components of the semi-wild and cultivated groups when K (the number of populations modeled) was set to 2. However, when K was set to 3, there were three different components, which also agreed well with the pattern in the phylogenetic tree. The sea island cotton and wild tetraploid cotton accessions were clustered as outgroup. Of them, the *G. darwinii* species exhibited closer relationships with sea island than the other two wild cotton species (*G. tomentosum* and *G. mustelinum*). However, the *G. tomentosum* species were more closely related to semi-wild upland cotton races than *G. darwinii* and *G. mustelinum*. All of the semi-wild cotton accessions were clustered into the semi-wild group, with the exception of only three accessions, *G. hirsutum* race *latifolium* accessions, which were clustered into the cultivated group. Phylogenetic analysis of semi-wild species further showed that on the whole, the semi-wild group could not be clearly separated according to the seven *G. hirsutum* races (**Supplementary Figure S2**). The landraces and modern cultivated cotton accessions were clustered into the cultivated group. With the exception of a small number of accessions that were close to semi-wild cotton, most of the landrace accessions were dispersed among modern cultivated accessions.

**Figure 2:**
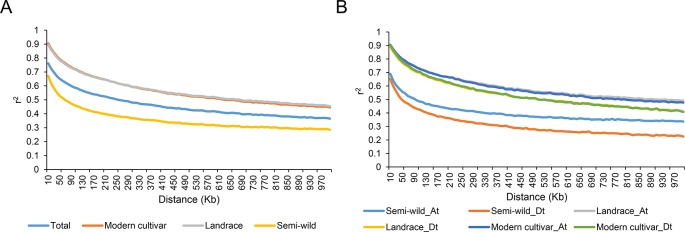
Decay of linkage disequilibrium (LD) in each group. **(A)**. Decay of LD in all samples. **(B)**. Decay of LD in sub-genome of each group.

The extent of linkage disequilibrium (LD) for each group was measured as the chromosomal distance when LD decreased to half of its maximum value. In the 416 cotton accessions, the average pairwise correlation coefficient (*r* *^2^*) dropped from 0.72 at 10 kb to 0.36 at 680 kb. The extent of LD in cotton was lower in the semi-wild group (420 kb) than in the cultivated groups (930 kb) (**Figure 2A**). For each group, the LD decay distance in the At sub-genome was higher than that in the Dt sub-genome. For example, the extent of LD in the semi-wild group was estimated to be 680 kb (*r* *^2^* = 0.16) in the At sub-genome and 300 kb (*r* *^2^* = 0.15) in the Dt sub-genome (**Figure 2B**).

### Selection Signals during Domestication and Improvement

We measured the genetic diversity of each group by calculating nucleotide diversity (π) values, and found that the genetic diversity of the semi-wild group (π = 1.25 × 10^−5^) was greater than that of the landrace group (π = 1.16 × 10^−5^). However, there was little difference between the landrace group and the modern cultivated group (π = 1.15 × 10^−5^) (**Supplementary Figure S3**). Further, population fixation statistics (*F*st) were investigated in the three groups. The *F*st value between the semi-wild group and the landrace group was 0.159 and the value between the landrace group and the modern cultivated group was 0.016 (**Supplementary Figure S3**).

**Figure 3:**
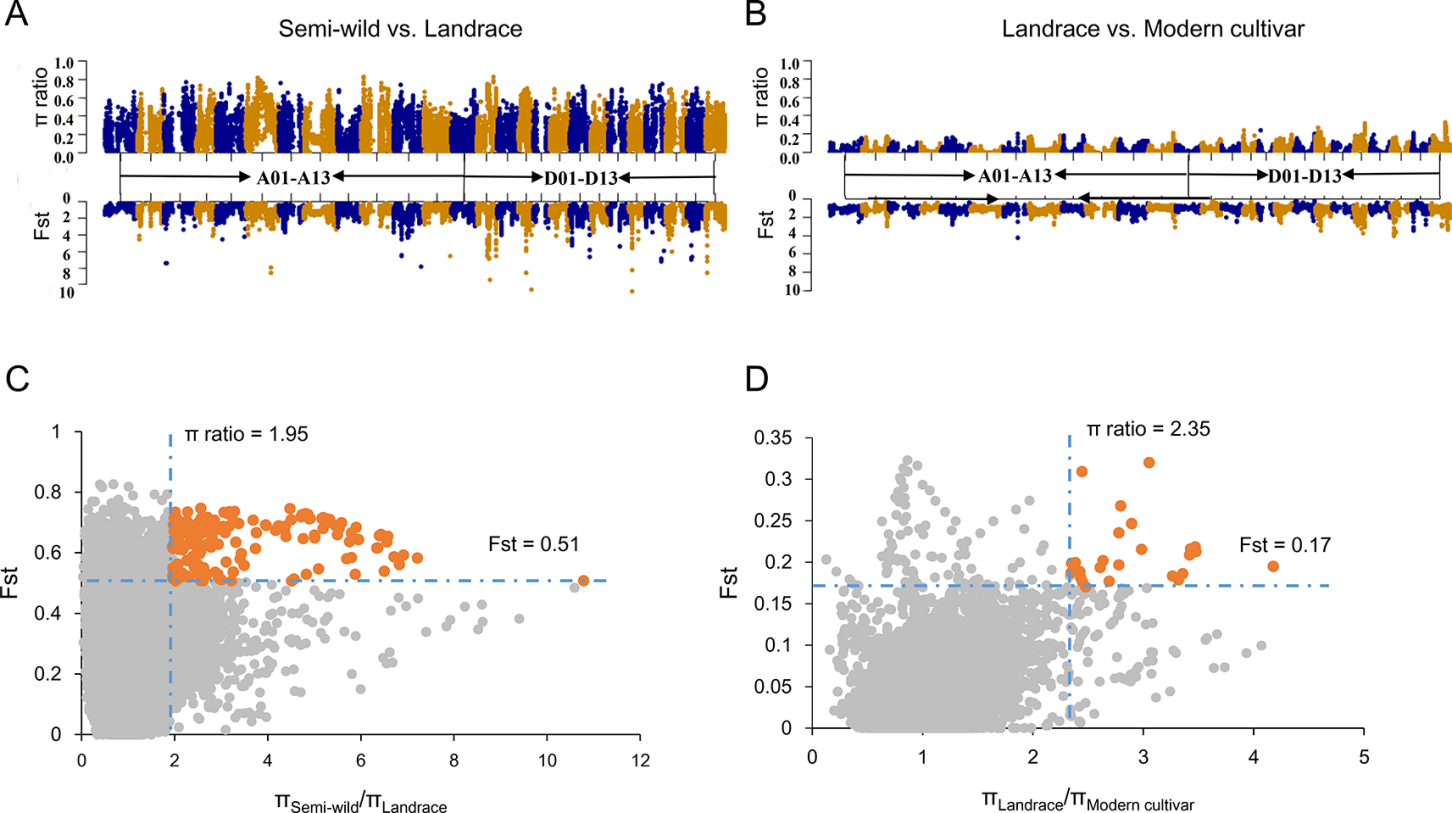
Identification of the selective sweeps in *G. hirsutum*. **(A)**. The values of π ratio and *F*st between semi-wild and landrace group were plotted against the position on each of the 26 chromosomes during domestication process. **(B)**. The values of π ratio and *F*st between landrace and modern cultivar group were plotted against the position on each of the 26 chromosomes during improvement process. **(C)**. Identification of the domestication selective sweeps. The orange dots indicated the selective sweeps. **(D)**. Identification of the improvement selective sweeps. The orange dots indicated the selective sweeps.

We integrated *F*st and ratio of π between each pairwise cotton group to identify potential selective signals during upland cotton domestication (semi-wild races versus landraces) and improvement (landraces versus modern cultivars). In order to ensure each sliding window had at least two SNPs, we counted the SNP distances and conducted a gradient simulation with different window sizes (**Supplementary Figure S4**). After combining the number of SNPs in the window with the LD of each cotton group, we identified that a suitable window size was 400 kb and the step was set as 40 Kb. As a result, a total of 71 DSS (**Figures 3A, C**) and 14 ISS (**Figures 3B, D**) were detected, which occupied 80.68 Mb (containing 2612 genes) and 13.04 Mb (containing 501 genes) on chromosomes, respectively (**Supplementary Tables S3**–**S5**). In addition, two ISS overlapped with the DSS on chromosomes A01 and D11, indicating a second round of selection (**Supplementary Table S3**). We found two GWAS signals involved in fiber elongation and micronaire (Ma et al., 2018), located on one of the overlapped selective sweeps (D11: 17.1-18.4 Mb) (**Supplementary Table S6**). Though the length of selective sweeps identified was close in each sub-genome, the π ratio and *F*st were higher in the Dt sub-genome, both in DSS and ISS (**Supplementary Figure S5**), implying that the Dt sub-genome experienced stronger selection during the two selection stages of upland cotton.

### Genes Relevant to Fiber Development in Selected Regions

Strong directional selection over the millennia was accompanied by transformation of the short, coarse, and brown fibers of wild plants into the long, strong, and fine white fibers of the modern cotton crop plant. To explore the evolutionary genetics of cotton fiber domestication, we overlapped selective sweeps with the locations of known GWAS loci associated with fiber yield and fiber quality, and found 4 and 82 GWAS signals during domestication, respectively. However, only 1 and 2 GWAS signals associated with fiber yield and fiber quality, respectively, were detected during improvement (**Supplementary Table S6**). This suggests that stronger artificial selection occurs during domestication than during improvement, and this is supported by the findings on population divergence (**Supplementary Figure S3**).

**Figure 4:**
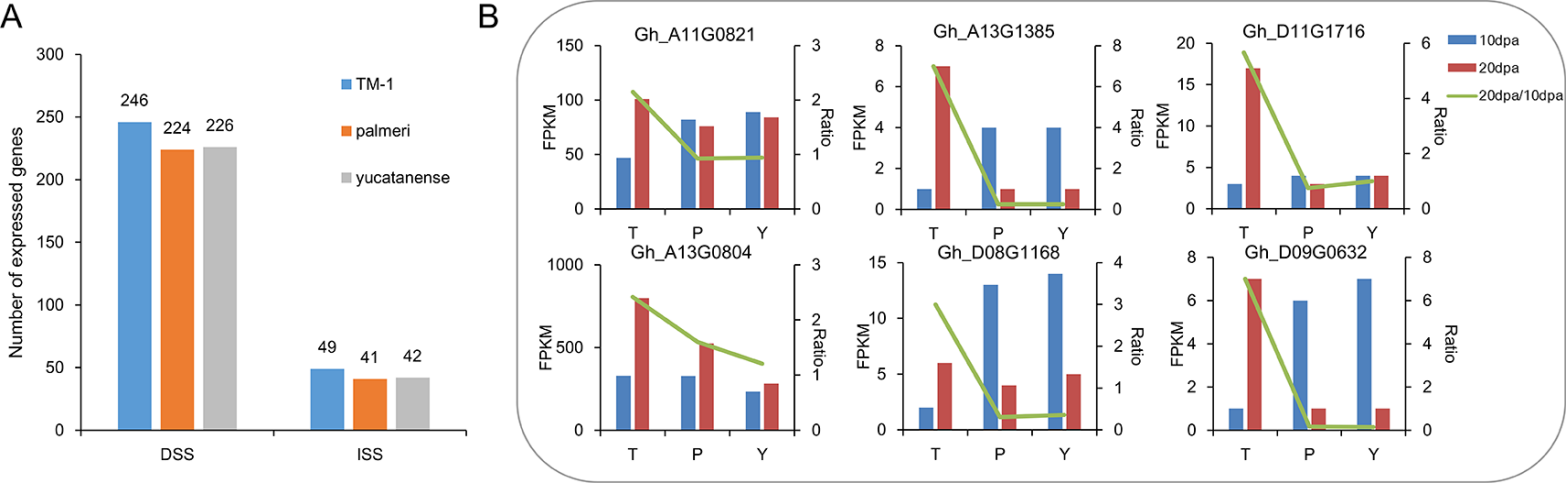
The number and expression patterns of genes related to fiber development in TM-1 and two semi-wild cotton relatives. **(A)**. The number of expressed genes during fiber development in TM-1, *palmeri* and *yucatanense*. **(B)**. The expression patterns of six genes related to fiber secondary cell wall development indicated early peak expression in semi-domesticated cotton races compared to TM-1. On the *x* axis, T indicates TM-1, P indicates *palmeri* and Y indicates *yucatanense*. The left *y* axis represents the FPKM value of each gene at 10 dpa and 20 dpa, and the right *y* axis represents the FPKM_20dpa_/FPKM_10dpa_ ratio.

To further investigate the contribution of selective sweeps to fiber development, a comprehensive profile of the TM-1 transcriptome that included multiple tissues was analyzed. In total, 255 and 53 genes were identified with predominant expression during fiber development in domestication and improvement sweeps, respectively (**Supplementary Table S7**, **Figure S6**). Of them, a large number of genes were related to fiber development, such as genes encoded MYB domain protein 60 (MYB60), RAB GTPase homolog 8 (RAB8), actin depolymerizing factor 1 (ADF1). We also compared the expression of these genes in TM-1 and two semi-wild cotton relatives (*G. hirsutum* race. *palmeri*; *G. hirsutum* race. *yucatanense*). Compared to TM-1, the number of expressed genes (FPKM > 3) was less in the two semi-wild cotton relatives in both DSS and ISS (**Figure 4A**, **Supplementary Table S8**). We found that Gh_D11G1128, which encodes ADP-ribosylation factor (ARF) was specifically expressed in cultivated upland cotton. Further, we selected the genes expressed predominantly at 20 and 25 dpa in TM-1 to investigate the difference in expression pattern between cultivated and semi-wild cotton relatives. Through fisher’s exact test (*p* < 0.05), we found that six genes (Gh_A11G0821, Gh_A13G0804, Gh_A13G1385, Gh_D08G1168, Gh_D11G1716, and Gh_D09G0632) related to secondary cell wall (SCW) development showed early peak expression that might lead to shorter fiber in semi-wild cotton relatives (**Figure 4B**, **Supplementary Table S8**).

### Genes Relevant to Stress Tolerance in Selected Regions

**Figure 5:**
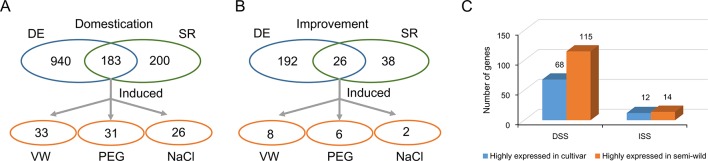
Identification of the genes related to stress response in selective regions. **(A)**. Identification of the genes related to stress response in DSS regions. **(B)**. Identification of the genes related to stress response in ISS regions. **(C)**. Statistics of the number of differentially expressed genes which annotated with GO term “response to stress.” The blue oval indicated the genes were differential expression between semi-wild and cultivated upland cotton group. The green oval indicated the genes annotated with GO term “response to stress.” The orange oval indicated the candidate genes overlapped both in blue and green ovals and were induced by different stress treatments in TM-1.

Domesticated species have an attenuated physiological stress response compared to their wild counterparts, but the genetic mechanisms underlying this change are not fully understood. To investigate the evolutionary characteristics of stress tolerance in upland cotton, we used GO analysis to identify 383 and 64 stress response related genes in DSS and ISS regions, respectively (**Supplementary Tables S9**, **S10**). Further, RNA-seq comparative analysis of semi-wild and cultivated cotton showed that 183 and 26 stress response genes were differentially expressed in DSS (**Figure 5A**) and ISS (**Figure 5B**), respectively. Of them, 62.8% (115/183) were down-regulated during domestication (Fisher’s exact tests, *p* = 0.02), and 53.8% (14/26) during improvement (Fisher’s exact tests, *p* = 1) (**Supplementary Tables S4**, **S5**). This implies that the decrease in stress tolerance of upland cotton mainly occurred during domestication (**Figure 5C**). To further investigate the function of these stress responsive genes, we performed transcriptome analysis on TM-1 with different stress treatments, including salt, drought, and *Verticillium dahliae*. In total, 46 genes in DSS regions, 32 of which were highly expressed in semi-wild accessions, were significantly induced by stresses, including 26 by salt, 31 by drought, and 33 against *Verticillium dahliae* challenge (**Figure 5A** and **Supplementary Table S4**, **Figure S7**). In ISS regions, 11 genes (6 highly expressed in semi-wild accessions) were induced by stresses, including two, six, and eight genes induced by salt, drought, and *Verticillium dahliae*, respectively (**Figure 5B** and **Supplementary Table S4**, **Figure S7**). Eight genes with high expression levels that were induced by two or more stresses were further screened, along with several genes previously reported to be related to plant stress responses (**Supplementary Table S11**).

**Figure 6:**
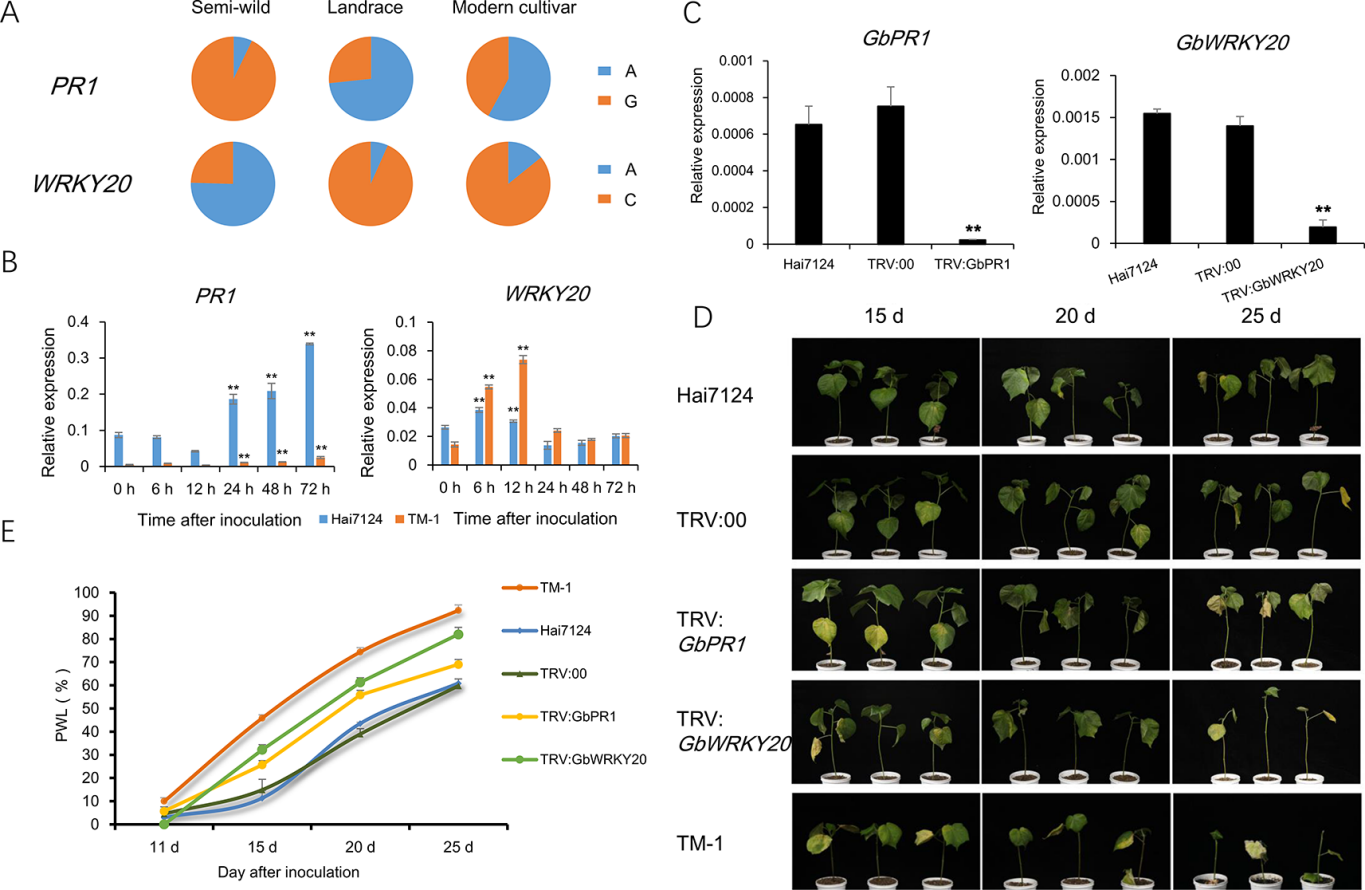
Functional validation of two candidate genes related to *Verticillium* Wilt resistance. **(A)**. The spectrum of allele frequencies at the causal polymorphisms of two genes in each upland cotton group. **(B)**. Relative expression levels of *PR1* and *WRKY20* were detected at six time point post inoculation in roots of TM-1 and Hai7124. *Ghhis3* was used as an internal control. Error bars represent the standard deviation of three biological replicates. ** indicate a significant difference to 0 h at *t*-test with *p* value of 0.01. **(C)** Expression of two genes in normal plant, injected with TRV::00 plant and silencing plant, detected by qRT-PCR in leaves of Hai7124. *Ghhis3* was used as an internal control. Error bars represent the standard deviation of three biological replicates. ** indicate a significant difference to normal plant and injected with TRV::00 plan at *t*-test with *p* value of 0.01. **(D)**. Plant phenotypic of each treatment in 15 d, 20 d and 25 d after V991 inoculation. **(E)**. The statistics of percentage of wilted leaves (PWL) in each treatment. Error bars represent the standard deviation of three replicates in each treatment.

The *PR* genes play important roles in signal recognition and plant immunity. Gh_A12G0274 encodes pathogenesis-related protein 1 (PR1), and the expression of *PR1* was about 60-fold higher in semi-wild cotton accessions than in modern cultivated accessions (**Supplementary Figure S8**). In addition, WRKY transcription factor family proteins play crucial roles in plant stress tolerance. Gh_D07G2328 encodes WRKY transcription factor family protein 20 (WRKY20). The expression of *WRKY20* was also higher in semi-wild cotton accessions than in modern cultivated accessions. Significant differences in allele frequencies at loci closely linked with these two genes were found between semi-wild and cultivated groups (**Figure 6A**). With Hai 7124 and TM-1 as controls resistant to and susceptible to *V. dahliae*, respectively, quantitative real time PCR (qRT-PCR) showed that *PR1* had significantly higher expression in Hai7124 compared with TM-1 and *WRKY20* had an earlier expression peak at 6 h post inoculation. Both genes were induced in Hai7124 and TM-1 after *V. dahliae* inoculation (**Figure 6B** and **Supplementary Figure S9**). VIGS confirmed that silencing *PR1* and *WRKY20* in Hai7124 plants significantly increases their susceptibility to *V. dahliae* (**Figures 6C–E**), indicating that these genes play a positive regulatory role in cotton disease resistance and their down-regulation in modern upland cotton cultivars compromised their resistance to *V. dahliae*.

## Discussion

Evolution and domestication of upland cotton has been extensively investigated through different methods, such as biochemical markers (Wendel et al., 1992), molecular markers (Brubaker and Wendel, 1994; Hinze et al., 2016), gene expression arrays (Hovav et al., 2008b), RNA-seq (Chaudhary et al., 2009; Yoo and Wendel, 2014), and whole genome resequencing (Fang et al., 2017a; Fang et al., 2017b; Wang et al., 2017; Ma et al., 2018). With the development of technology, the high-quality upland cotton genome has been released (Li et al., 2015; Zhang et al., 2015; Hu et al., 2019; Wang et al., 2019) and several reports published on elucidating the evolutionary characteristics and mechanisms of upland cotton (Fang et al., 2017a; Fang et al., 2017b; Wang et al., 2017; Ma et al., 2018).

In present study, we found that the *G. tomentosum* species were closely related to semi-wild upland cotton races which is consistent with that of a previous report (Wendel and Cronn, 2003). Three *G. hirsutum* race *latifolium* accessions were clustered into the cultivated group which supports the theory that domesticated cultivated upland cotton mainly originated from *G. hirsutum* race *latifolium* (Brubaker and Wendel, 1994). The LD decay distances in this study is similar to the findings of Fang et al. (2017b), but lower than values reported by Wang et al. (2017). Comparing to other crops, the LD decay distances for cotton are much higher than soybean (Lam et al., 2010) and rice (Xu et al., 2011), but lower than *Brassica* (Zou et al., 2018). Phenotypic traits that are favorably selected by humans to enhance agricultural characteristics usually have low levels of variation and skewed allele frequency spectra (Tajima, 1989). In addition, *F*st is not strongly affected by ascertainment bias, therefore it is better suited to analyze data generated by SNP chips (Albrechtsen et al., 2010).Our analysis showed that the *F*st value between semi-wild and landrace accessions was much greater than that between landrace and modern cultivated upland cotton accessions, which is consistent with previous reports (Fang et al., 2017a; Fang et al., 2017b). In addition, the *F*st in cotton was higher than that in rice (Xu et al., 2011) and sesame (Wei et al., 2015), but lower than that in soybean (Zhou et al., 2015). The analysis of population characteristics suggests that there is a remarkably similar genetic background between landraces and modern cultivars. This may be because the domestication process from semi-wild races to landraces lasted for thousands of years, while the improvement process from landraces to modern cultivated species occurred over a much shorter time period (Wendel et al., 1992; Tyagi et al., 2014). It also indicates that these landraces contributed to the development of different breeding populations during the improvement of modern varieties (Fang et al., 2017b). Furthermore, the genetic diversity of upland cotton is smaller than that of other crops, and cotton breeding has been frequently involved crossing and re-selection within small sets of breeding materials, which has led to the loss in genetic diversity (May et al., 1995). In addition, no clear boundary existed among the seven races in the semi-wild population. Other researchers have also reported high levels of introgression within the semi-wild accessions (Hinze et al., 2016).

In addition to its economic value, tetraploid cotton is an outstanding model system for studying cell elongation, cell wall biosynthesis, and polyploidization (Malik et al., 2014). The evolution and domestication process of cotton is more complex than other polyploidy crops. Most researches have focused on the development of cotton fibers during the process of polyploidization and later evolution (Hovav et al., 2008a; Hovav et al., 2008b); however, there are still many puzzles about the evolution and domestication of upland cotton. In this study, selective sweeps that occurred during domestication and improvement were identified, representing two stages of artificial selection. We found that artificial selection was unequal in these two stages, and the process of domestication selection (from semi-wild to landraces) was more intense. In addition, the artificial selection had bias for the D sub-genome. In selective sweeps, more fiber development related genes were located on the D sub-genome than on the A sub-genome. At the transcriptional level, D sub-genome expression was preferentially enhanced under human selection pressure, compared to homeologous genes on the A sub-genome (Hovav et al., 2008a; Hovav et al., 2008b). This indicates that the superior fibers of modern upland cotton are closely related to artificial selection for D sub-genome genes. In recent reports, Wang et al. (2017) found most QTL hotspots related to fiber development that overlapped with selection sweeps during domestication, were located in the D sub-genome. In addition, scanning electron micrograph analysis of ovules also showed that genes for fiber improvement in tetraploid cotton were contributed by the agronomical inferior D genome diploid parent (Applequist et al., 2001). In this study, we found that an important fiber development-related gene ARF was located in D sub-genome. ARFs are essential for vesicle coating and uncoating in all eukaryotic cells, which is involved in regulating membrane trafficking and cytoskeleton rearrangements at the plasma membrane by cycling between the GTP-bound active and GDP-bound inactive conformations (Naramoto et al., 2010). Silencing ARFs can reduce the amount of cellulose in cell walls and affects cell size in Arabidopsis (Gebbie et al., 2005). Ras/ARF proteins have been reported to be over-represented through positive selection (*Ka*/*Ks* >1) in modern cultivated cotton (Wang et al., 2019). In addition, several genes in selective sweeps might play crucial roles in fiber development, such as *MYB60* (Kasirajan et al., 2018), *RAB8* (Li and Guo, 2017), and *ADF1* (Zhang et al., 2007).

It is widely considered that fiber elongation terminates at about 2 weeks post anthesis in most wild cotton species while growth is extended to three or more weeks in cultivated cotton species (Applequist et al., 2001; Hu et al., 2019). This is caused by high concentrations of reactive oxygen species (ROS), leading to the early initiation of secondary-cell-wall synthesis and consequent prevention of rapid fiber elongation in wild cotton fibers (Guo et al., 2016; Wang et al., 2017). In the present study, we found that the expression peaks of several secondary cell wall synthesis related genes were in earlier fiber development stages in semi-wild upland cotton than in modern cultivars.

Conventional breeding to domesticate wild plants increases productivity but is often accompanied by decreased fitness and genetic diversity, thus hampering growth in challenging environmental conditions (Meyer and Purugganan, 2013). Long-term domestication selection and modern breeding techniques have significantly improved cotton fiber yield and quality. However, breeding of cotton over millennia has also led to reduced genetic diversity. As a result, beneficial traits of wild species, such as disease resistance and abiotic stress tolerance, have been lost. With the decrease in arable land area and the deterioration of soil environments throughout the world, the demand for stress tolerance is becoming increasingly urgent. The improvement of the stress tolerance of crops has become the focus of breeders. Therefore, a major objective in modern cotton breeding is to restore alleles from wild ancestors of crop plants which were ‘‘left behind’’ during domestication and may be useful for the improvement of productivity under stress conditions. In present study, we found most stress response genes were down-regulated during domestication, and this is responsible for the decreased stress tolerance of upland cotton (**Figure 5**). *PR1* gene is a useful molecular marker for the salicylic acid (SA) response and has been reported to play crucial roles in systemic acquired resistance in *Arabidopsis* (Li and Kang, 2018). VIGS assay showed that silencing of *PR1* significantly reduced the disease resistance of cotton (**Figure 6**). Furthermore, we found that the expression of *PR1* in semi-wild cotton was about 60 times higher than that of modern cultivated cotton (**Supplementary Table S9**), implying the down-regulated expression of *PR1* and compromised disease resistance due to the process of domestication from semi-wild to cultivated accessions. *WRKY20* have been reported playing crucial roles in plant stress tolerance (Luo et al., 2013; Yan et al., 2017). Overexpression of *GbWRKY1* (homologs of *WRKY20*) from sea island cotton can positively regulate the Pi starvation response by alteration of auxin sensitivity in *Arabidopsis* (Xu et al., 2012). In this study, we found that silencing of *WRKY20* significantly reduced disease resistance in sea island cotton.

During crop domestication, stress resistance is traded for yield potential. High expression of stress tolerance genes is not conducive to crop growth and development, possibly due to resource competition in cells or because high expression of some stress-resistant genes can inhibit plant growth, for example, ABA can control stoma closure and impedes the process of photosynthesis (Tanaka et al., 2013). In rice, simultaneously mutating the genes encoding the ABA receptors pyrabactin resistance 1-like 1 (PYL1), PYL4, and PYL6, causes improved growth and increased grain yield, however, plants will be more sensitive to drought stress (Miao et al., 2018). Early breeding efforts focused on high crop yields, which led to reduced stress resistance in many modern varieties, such as rice (Lachagari et al., 2019) and maize (Mao et al., 2015). Evolution has created thousands of species of naturally stress-resistant plants, most of which are traditional landraces or wild relatives of modern cultivated species (Zhang et al., 2018). With the development of technology, such as high-throughput sequencing, genome editing, and plant transformation, the naturally stress-resistant plants can be utilized for the rapid genetic improvement of modern varieties. In this study, based on the identified domestication and artificial selection regions, we found that the semi-domesticated cotton accessions can provide elite genes for the increased stress tolerance and stable productivity of modern cotton cultivars.

## Data Availability Statement

The RNA-seq data of different stress treatments with drought, salt, and *Verticillium dahliae*, respectively, in TM-1 have been deposited in the National Center for Biotechnology Information (NCBI) database under BioProject accession PRJNA532694.

## Author Contributions

WG conceived this project. WG and GZ designed all experiments. GZ performed the experiments and analyzed the data under the supervision of WG, with assistance from WL and LL for bioinformatics analyses, GW and QS for VIGS analysis, and CC for SNP chip analysis. WG and GZ wrote the manuscript, and WG revised the manuscript. All authors discussed the results and commented on the manuscript. All authors read and approved the final manuscript.

## Funding

This work was supported by the National Transgenic Program (2018ZX0800918B), National Key R and D Program for Crop Breeding (2016YFD0100306; 2018YFD0100401), Jiangsu Collaborative Innovation Center for Modern Crop Production project (No. 10) and China Postdoctoral Science Foundation (2019M651856). The funders had no role in study design, data collection and analysis, decision to publish, or preparation of the manuscript.

## Conflict of Interest

The authors declare that the research was conducted in the absence of any commercial or financial relationships that could be construed as a potential conflict of interest.
