# Supplementary material for: Genetic Basis of Fiber Improvement and Decreased Stress Tolerance in Cultivated Versus Semi-Domesticated Upland Cotton
Source: Front Plant Sci. 2019 Nov 29;10:1572. doi: 10.3389/fpls.2019.01572 (PMC6895062; doi:10.3389/fpls.2019.01572)
Supplement: Supplementary file 1 [file DataSheet_1.zip › Supplemental_information_figures.docx]

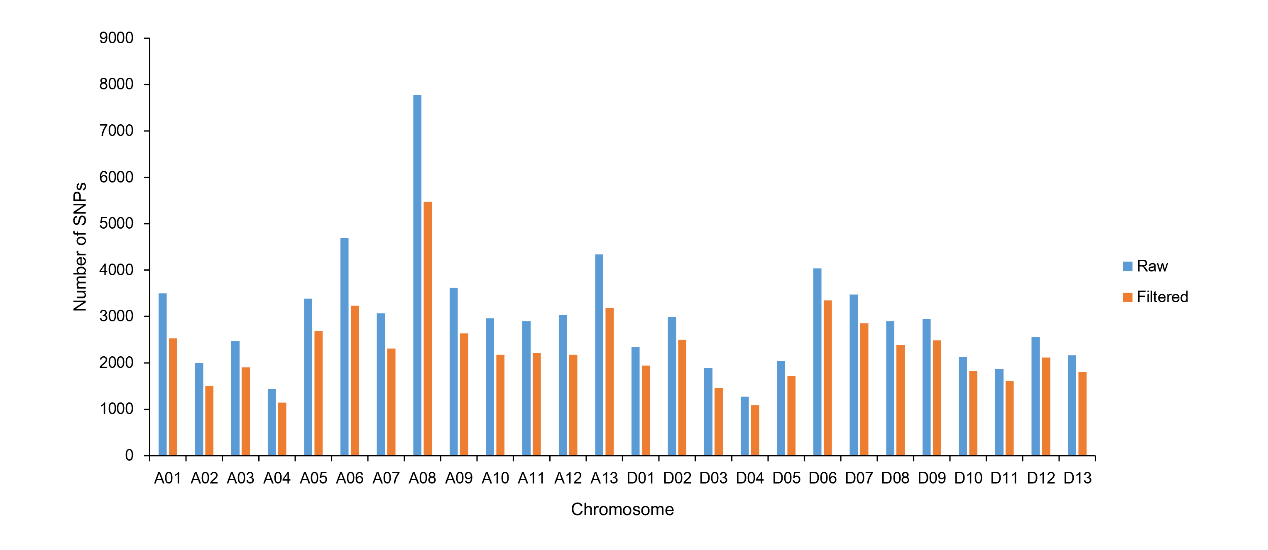


**Figure S1.** Chromosome distribution of SNPs from CottonSNP80K.

**
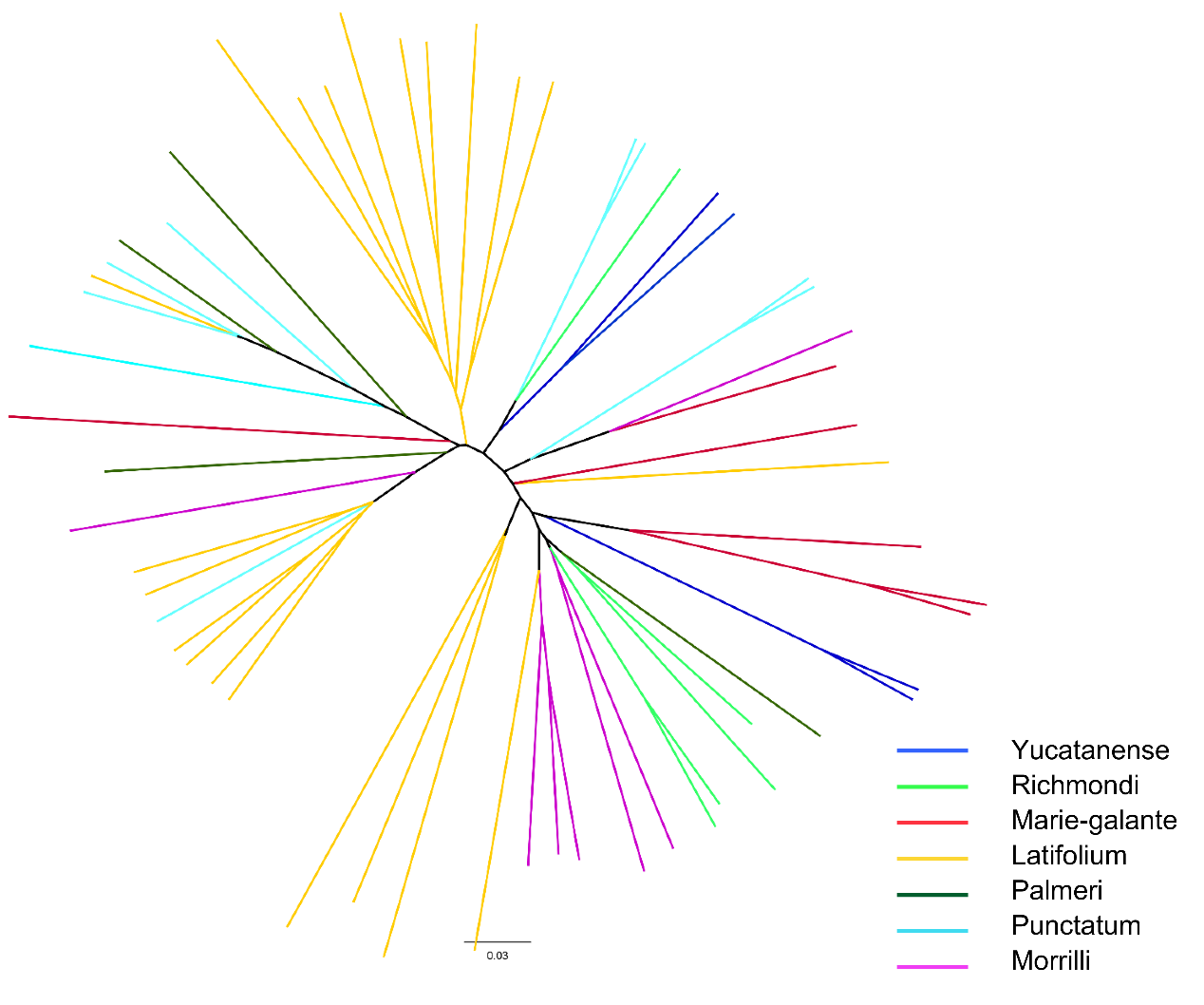
**

**Figure S2.** A neighbor-joining tree of semi-wild cotton using SNP data from CottonSNP80K.

**
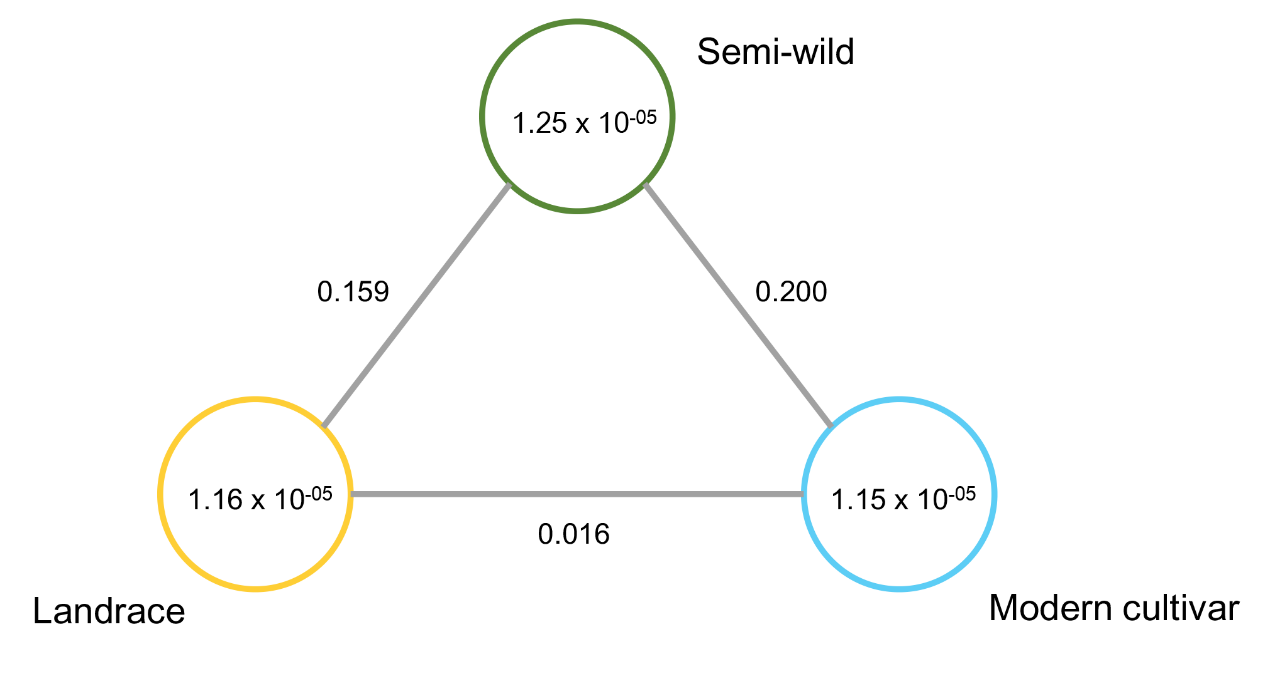
**

**Figure S3.** Nucleotide diversity (π) and population divergence (*F*st) across the semi-wild, landrace and modern cultivated cotton groups. The value in each circle represents nucleotide diversity for this group, and the value on each line indicates population divergence between the two groups.

**
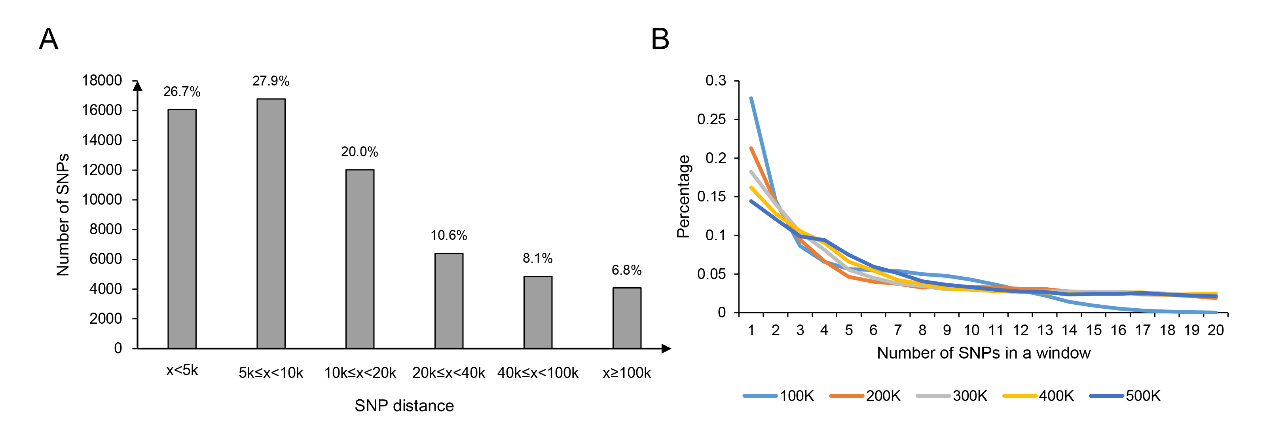
**

**Figure S4.** Evaluation of windows for the calculation of nucleotide diversity (π) and population divergence (*F*st). **A.** Statistics of SNPs distance used in present study. **B.** SNP content of windows for different window size.

**
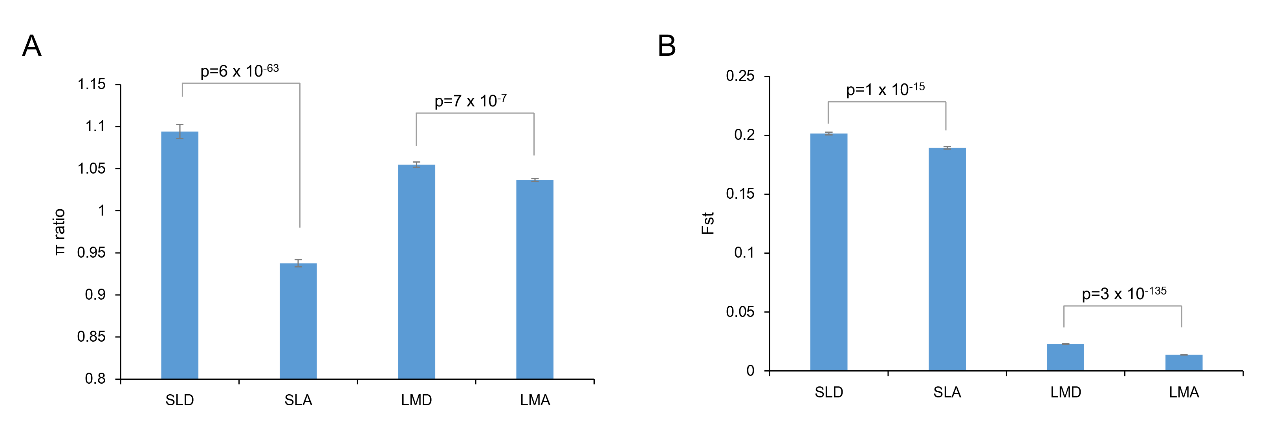
**

**Figure S5.** Statistics of π ratio and *F*st between At and Dt sub-genome. **A.** The statistics of π ratio. **B.** The statistics of *F*st. SLD: compared semi-wild to landrace group in Dt sub-genome. SLA: compared semi-wild to landrace group in At sub-genome. LMD: compared landrace to modern cultivar group in Dt sub-genome. LMA: compared landrace to modern cultivar group in At sub-genome.

**
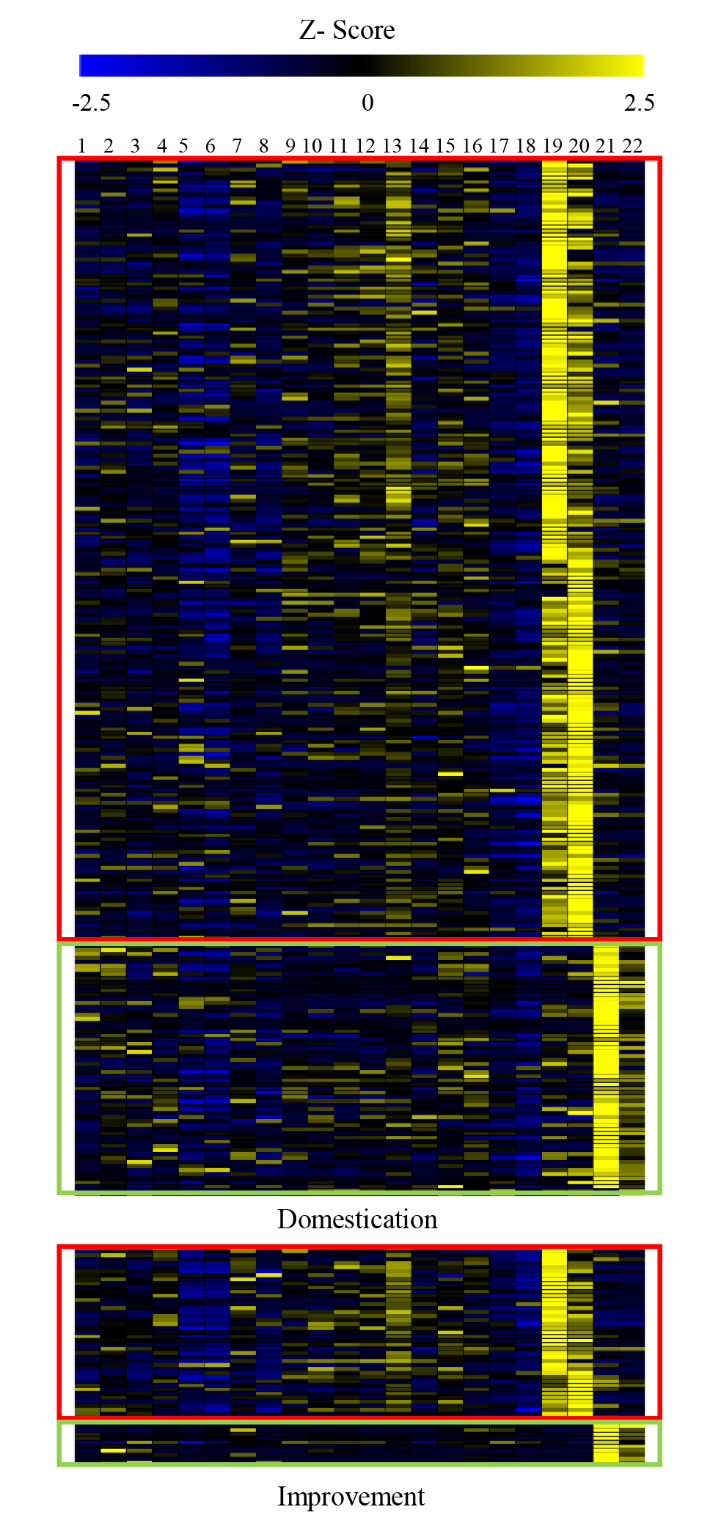
**

**Figure S6.** Heatmap of predominantly expressed genes in cotton fiber. The red box indicated high expression during fiber elongation period. The green box indicated high expression during secondary wall thickening period. The tissues or organs were marked as: 1, root; 2, stem; 3, leaf; 4, torus; 5, petal; 6, stamen; 7, pistil; 8, calycle; 9, -3dpa ovule; 10, -1dpa ovule; 11, 0dpa ovule; 12, 1dpa ovule; 13, 3dpa ovule; 14, 5dpa ovule; 15, 10dpa ovule; 16, 20dpa ovule; 17, 25dpa ovule; 18, 35dpa ovule; 19, 5dpa fiber; 20, 10dpa fiber; 21, 20dpa fiber; 22, 25dpa fiber, respectively.

**
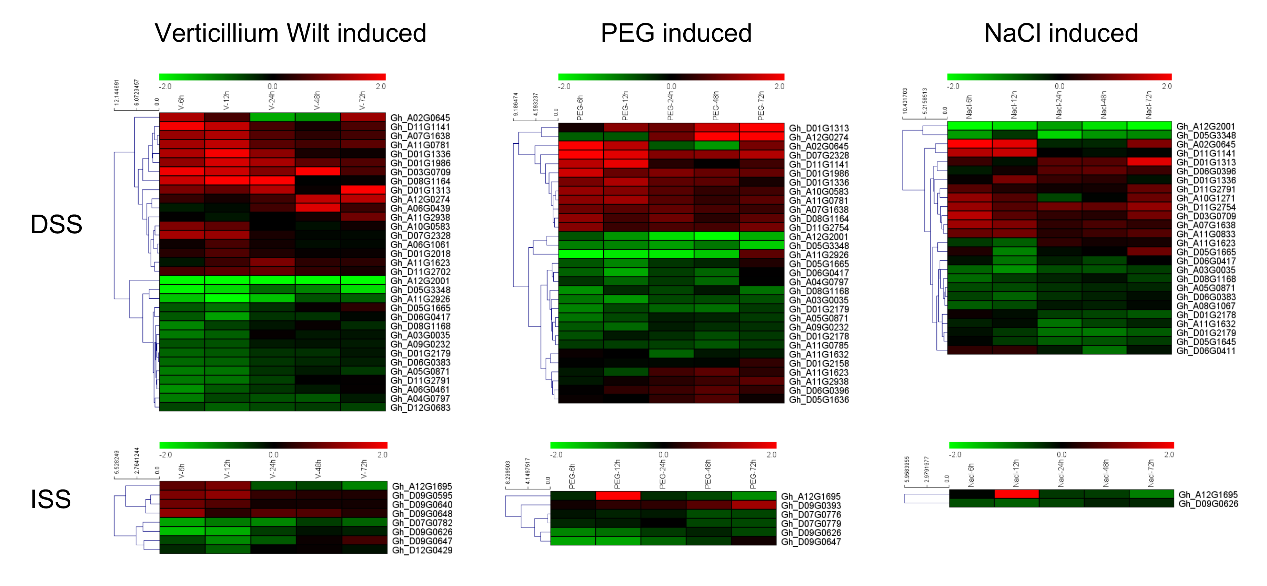
**

**Figure S7.** Induced expression pattern of genes related to stress response in DSS and ISS by *Verticillium dahliae*, PEG and NaCl, respectively. Expression data for each treatment were from three biologically independent experiments, respectively.

**
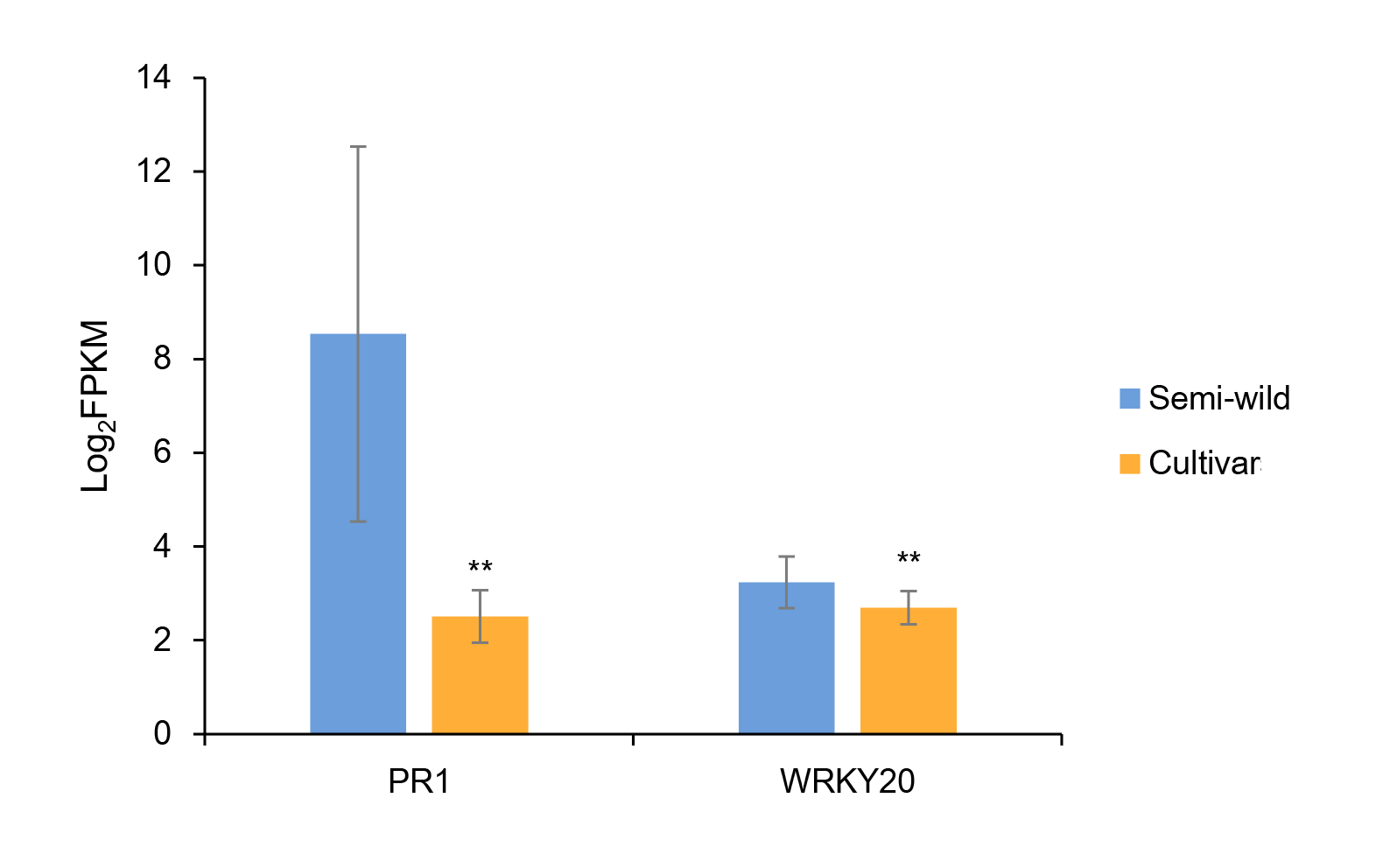
**

**Figure S8.** Expression level of *PR1* and *WRKY20* in semi-wild and cultivars. Error bars represent the standard deviation of gene expressive abundance in 10 semi-wild and 40 cultivated cotton accessions, respectively. ** indicate a significant difference at *t*-test P-value of 0.01.

**
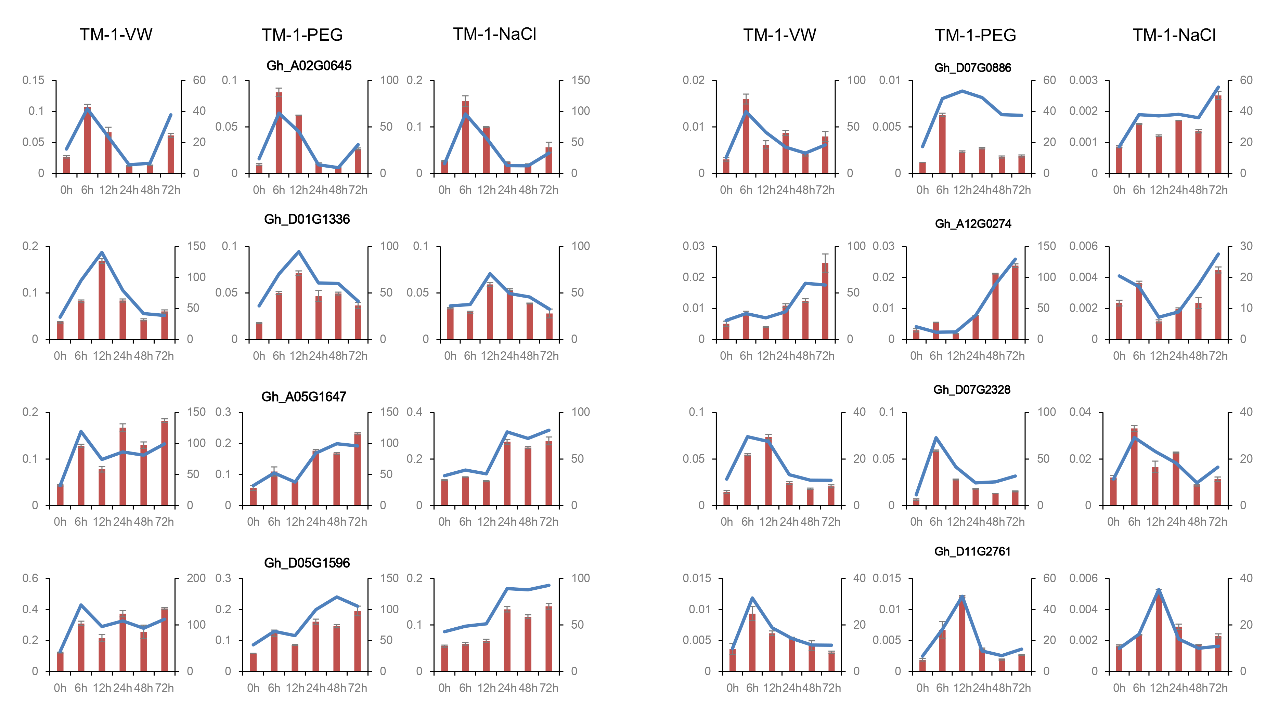
**

**Figure S9.** Expression pattern of eight candidate genes involved in three kinds of stresses tolerance. The x-axis represents the time post stress treatment. The left y-axis: The red bars indicated the relative expression of each gene at different time point post stress treatment using qRT-PCR. The right y-axis: The blue lines indicated the FPKM of each gene at different time point post stress treatment. Expression data for each treatment were from three biologically independent experiments, respectively. Error bars represent the standard deviation of three biological replicates in qRT-PCR assay.
